# Epidemiological surveillance of canine visceral leishmaniasis in a municipality in the Western Amazon

**DOI:** 10.1590/S1678-9946202668063

**Published:** 2026-09-18

**Authors:** Amanda Carolina Barbosa de Aguiar, Leormando Fortunato Dornelas, Eric Fabrício Marialva, Andre de Abreu Rangel Aguirre, Claudia María Ríos Velásquez, Felipe Arley Costa Pessoa, Hemelly Suldini da Silva, Carolina Bioni Garcia Teles, Amália dos Santos Ferreira, Raiane Gosenheimer Peruffo, Ivaneide Nunes da Costa Silva, Isis Hemanuely Suldini Padilha, Janaina Rodrigues Raiser, Monizi Angélica Alves Steger, Gabriel de Sá Pitangui Almeida, Kamila Pereira de França, Lilith Helena de Castro Barroso, Sergio de Almeida Basano, Luis Marcelo Aranha Camargo

**Affiliations:** 1 Universidade de São Paulo, Faculdade de Medicina Veterinária e Zootecnia, Programa de Pós-Graduação em Epidemiologia e Saúde Pública, São Paulo, São Paulo, Brazil; 2 Universidade de São Paulo, Instituto de Ciências Biomédicas, Monte Negro, Rondônia, Brazil; 3 Instituto Federal de Educação, Ciência e Tecnologia de Rondônia, Campus Jaru, Jaru, Rondônia, Brazil; 4 Instituto Nacional de Epidemiologia da Amazônia Ocidental, Porto Velho, Rondônia, Brazil; 5 Instituto Leônidas e Maria Deane, Manaus, Amazonas, Brazil; 6 Fiocruz Rondônia, Laboratório de Entomologia, Porto Velho, Rondônia, Brazil; 7 Diagnostika Centro de Diagnóstico Animal, Ariquemes, Rondônia, Brazil; 8 Programa de Pós-Graduação em Biodiversidade e Biotecnologia da Amazônia Legal, Porto Velho, Rondônia, Brazil; 9 Fiocruz Rondônia, Plataforma de Bioensaios de Malária e Leishmaniose, Porto Velho, Rondônia, Brazil; 10 Santo Chico Serviços Veterinários, Monte Negro, Rondônia, Brazil; 11 Centro Universitário Aparício Carvalho, Porto Velho, Rondônia, Brazil; 12 Centro de Medicina Tropical de Rondônia, Porto Velho, Rondônia, Brazil; 13 Centro de Pesquisa em Medicina Tropical de Rondônia, Porto Velho, Rondônia, Brazil; 14 Centro Universitário FAEMA, Ariquemes, Rondônia, Brazil; 15 Universidade Federal do Acre, Laboratório de Medicina Tropical, Rio Branco, Acre, Brazil

**Keywords:** Visceral leishmaniasis, Dogs, Sand flies, One Health, Amazonia

## Abstract

Visceral leishmaniasis is a zoonotic disease of public health importance in Brazil, with expansion into peri-urban areas. This cross-sectional study evaluated 450 dogs in a municipality of the Western Amazon, Brazil, using TR-DPP^®^ serology, ELISA confirmation, hematological analysis, PCR targeting the *Hsp70* gene, and entomological surveys with CDC light traps. A total of 45 dogs (10%) were seroreactive, and 34 were confirmed by ELISA. Hemoparasites such as *Babesia* spp*.* were detected. All pooled samples were negative by PCR. Six species of phlebotomine sand flies were identified, including *Nyssomyia antunesi* and *Migonemyia migonei*. The findings suggest early exposure of dogs in an area of emerging epidemiological risk, highlighting the importance of integrated surveillance strategies.

## INTRODUCTION

Visceral leishmaniasis (VL) is a severe zoonotic disease of major public health importance in Brazil, characterized by chronic progression and potentially fatal outcomes when left untreated. Historically associated with rural and sylvatic environments, VL has undergone an intense urbanization process since the second half of the twentieth century, driven by environmental modifications, unplanned urban expansion, population migration, and the adaptation of vectors to anthropized areas^1^ ^,^ ^2^.

Dogs play a central role in the transmission cycle of visceral leishmaniasis, acting as the main urban reservoirs of *Leishmania infantum*. Canine infection frequently precedes human cases, making dogs important sentinel indicators for epidemiological surveillance. Consequently, areas with evidence of canine infection, even in the absence of confirmed human cases, should be considered priorities for surveillance and control actions, especially in regions classified as emerging or silent transmission areas^3^ ^,^ ^4^.

In Brazil, VL transmission exhibits marked regional heterogeneity influenced by ecological and environmental factors affecting vector distribution and abundance. In the Amazon region, processes such as deforestation, urban expansion, and habitat fragmentation have intensified interactions among vectors, reservoirs, and human populations, contributing to the emergence of new transmission scenarios^5^ ^-^ ^9^.

The transmission dynamics of *Leishmania* involve complex interactions among vectors, reservoirs, hosts, and environmental conditions, particularly in regions undergoing rapid ecological transformation^10^. These processes favor the establishment and maintenance of transmission cycles in areas affected by urban growth and environmental disturbance^11^ ^,^ ^12^.

Although Rondonia State has historically been considered free of autochthonous visceral leishmaniasis transmission, recent studies have demonstrated the circulation of *Leishmania infantum* in dogs from municipalities such as Ji-Parana and Cacoal, in addition to the presence of competent vectors in urban and peri-urban environments^1^ ^,^ ^13^ ^,^ ^14^.

Furthermore, reports of canine visceral leishmaniasis in Porto Velho involving animals without a history of travel to endemic areas suggest the possibility of local transmission^14^. Together, these findings indicate increasing epidemiological vulnerability and the potential emergence of new transmission foci in the region.

Despite this growing evidence, studies integrating data on canine infection, vector occurrence, and local environmental conditions in small municipalities of inland Rondonia remain scarce. This gap limits the understanding of the early establishment of visceral leishmaniasis transmission in areas that may be characterized as silent transmission zones^13^ ^,^ ^15^.

Therefore, this study aims to evaluate the epidemiological scenario of canine visceral leishmaniasis in Monte Negro municipality, Rondonia, using integrated serological, molecular, hematological, and entomological approaches.

### Ethics

All procedures involving animals were conducted in accordance with current ethical standards for the use of animals in research, as established by the Brazilian National Council for the Control of Animal Experimentation (CONCEA). The study was approved by the Ethics Committee on the Use of Animals of the Institute of Biomedical Sciences of the University of Sao Paulo (CEUA-ICB/USP), under protocol Nº 1290291123 (ID 053138), with an amendment approved on May 13, 2025.

## MATERIALS AND METHODS

### Study area

**Figure 1:**
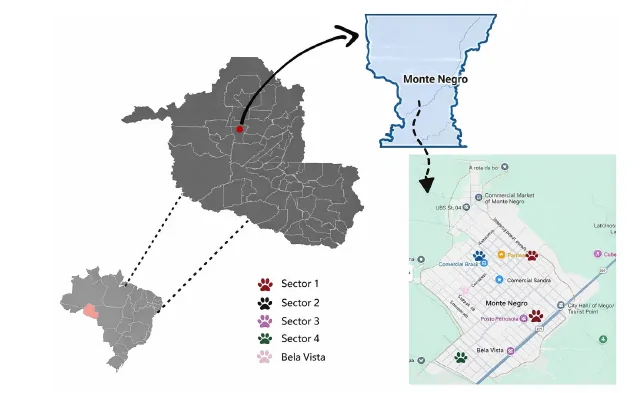
Neighborhoods of Monte Negro where dog blood samples were collected, 2024-2025.

The study was conducted in Monte Negro municipality, located in Rondonia State, Brazil (10°15’37.8”S, 63°17’6.3”W). The municipality has an estimated population of 12,168 inhabitants, of whom approximately 5,520 live in the urban area, according to estimates from the Brazilian Institute of Geography and Statistics (IBGE)^16^. A total of 450 dogs, domiciled and stray, were included in the study and were sampled across neighborhood sectors 1, 2, 3, 4, and Bela Vista, which are representative areas of the urban zone of the municipality. Data were collected from February 2024 to May 2025 (Figure 1).

### Canine sampling and serology

A total of 450 blood samples were collected from domiciled and stray dogs in different urban sectors of Monte Negro municipality, Rondonia State. For spatial epidemiological analysis, the urban area of the municipality was stratified into sectors according to the local territorial designation adopted during field sampling. Sample collection was performed by jugular and/or cephalic vein puncture, yielding approximately 5 mL of blood per animal, which was placed into tubes containing EDTA and plain tubes.

Based on the technical guidelines of the Brazilian Ministry of Health, an approximate ratio of one dog per 6.6 inhabitants is estimated; therefore, the urban canine population of Monte Negro is estimated at approximately 1,840 dogs. Thus, the sample evaluated in the present study corresponded to approximately 24.4% of the estimated urban canine population, representing substantial coverage for epidemiological purposes, according to the protocol of the Brazilian Ministry of Health^17^.

Samples collected in plain tubes were centrifuged at 5,000 rpm for 10 min to separate the serum, which was aliquoted into three fractions and stored at −20 °C in DNA-free PVC tubes. The first aliquot was used to perform the TR-DPP^®^ rapid serological test (Bio-Manguinhos/FIOCRUZ), according to the manufacturer’s recommendations.

For blood samples collected in tubes containing anticoagulant, automated complete blood counts were performed using an IDEXX^®^ analyzer. Blood smears were prepared on glass slides, stained with Giemsa, and examined under optical microscopy (Zeiss^®^) at 1,000× magnification (under oil immersion) for the detection of hemoparasites. Identification of *Babesia* spp*., Ehrlichia canis,* and other hemoparasites was performed based on morphological characteristics, including intraerythrocytic forms and leukocyte inclusions, according to established diagnostic criteria. During household visits, animal owners or caretakers completed an epidemiological questionnaire containing information on household characteristics, proximity to wooded areas, family or canine history of visceral leishmaniasis, age, sex, vaccination status, and the presence of clinical signs compatible with the disease. At the time of sample collection, informed consent was obtained from the animal owner or guardian. 

### Molecular analyses

Blood samples from dogs reactive in the TR-DPP^®^ immunochromatographic test were subsequently analyzed by enzyme-linked immunosorbent assay (ELISA) (EIE-LVC^®^, Bio-Manguinhos/FIOCRUZ), performed according to the manufacturer’s instructions. This assay was used as a confirmatory serological test for the detection of anti-*Leishmania* spp. IgG antibodies.

Optical density was measured using a microplate reader, and samples with values above the cutoff established by the kit were considered positive.

Molecular analysis by PCR was conducted at the Oswaldo Cruz Foundation (Fiocruz) Entomology Laboratory in Rondonia. Genomic DNA was extracted from blood clot samples preserved in 70% ethanol using a protocol based on proteinase K digestion and SDS-mediated lysis, followed by organic extraction and ethanol precipitation. Briefly, 70-100 mg of blood clot was incubated with proteinase K (20 mg/mL) and 20% SDS at 65 °C for 1 h. DNA purification was performed by chloroform extraction, followed by precipitation with cold ethanol and subsequent washing steps. The DNA pellet was resuspended in TE buffer containing RNase and stored at −30 °C until use. 

Given the large number of samples, DNA extracts were organized into pools containing up to five samples, totaling 103 pools. This strategy was adopted to optimize large-scale screening; however, it may reduce analytical sensitivity, particularly in samples with low parasitic load.

Detection of *Leishmania* DNA was performed by polymerase chain reaction (PCR), targeting the *Hsp70* gene, using the primers HSP70-240F (5’-GGACGAGATCGAGCGCATGGT-3z’) and HSP70-240R (5’-TCCTTCGACGCCTCCTGGTTG-3’), which amplify a fragment of approximately 240 bp, as described by Graça *et al*.^18^. PCR reactions were carried out in a final volume of 25 μL, containing 12.5 μL of GoTaq Green Master Mix (Promega), 1.0 μL of each primer, 5.5 μL of ultrapure water, and 5 μL of template DNA.

Amplification was performed in a thermocycler under the following conditions: initial denaturation at 94 °C for 5 min; followed by 35 cycles of denaturation at 94 °C for 30 s, annealing at 61 °C for 30 s, and extension at 72 °C for 45 s; with a final extension at 72 °C for 10 min.

Genomic DNA of *Leishmania infantum* was used as a positive control, and ultrapure water as a negative control. To assess potential PCR inhibition, internal amplification controls were used in a subset of samples**,** reducing the likelihood of false-negative results.

PCR products were analyzed by agarose gel electrophoresis stained with ethidium bromide and visualized under ultraviolet light.

### Entomological study

Entomological collections were carried out in two main types of environments: the peridomicile, defined as the immediate surroundings of residences, including backyards, shelters for domestic animals, and breeding areas; and forested areas, corresponding to forest fragments adjacent to households. This categorization allowed comparison of phlebotomine occurrence in domestic and nearby sylvatic environments.

Phlebotomine sand flies were captured using CDC light traps. The traps remained active for 72 consecutive hours and were operated for three consecutive days during each monthly sampling campaign, preferably during new moon periods, at a height of 1.5 m above ground level, characterizing a standardized sampling effort throughout the study.

After collection, the insects were sent to the ICB-5/USP, Rondonia Unit, located in Monte Negro municipality, where initial screening and preliminary identification were performed under a Zeiss^®^ stereomicroscope at 40× magnification, aiming to separate phlebotomines from other arthropods. Subsequently, specimens were individually placed in vials containing absolute ethanol and kept under refrigeration to prevent alcohol evaporation.

The entomological material was then sent to the Laboratory of Ecology of Transmissible Diseases in the Amazon at ILMD/FIOCRUZ Amazonia, in Manaus, Amazonas State, where taxonomic identification of species and sex determination were performed. Taxonomic identification of captured phlebotomine sand flies was based on external morphological characteristics and genital structures. Specimens were previously cleared and mounted on microscope slides to allow observation of characters such as male terminalia, female spermathecae, cibarium, pharynx, and wing venation, following criteria widely used in medical and veterinary entomology. Species determination was carried out using specialized taxonomic keys, including morphological approaches described by Galati and collaborators and previously established illustrated keys for Neotropical *Phlebotominae*, which encompass the main diagnostic characters of males and females^19^.

### Follow-up of owners of seropositive dogs

In households with seropositive dogs (TR-DPP^®^), residents were examined and invited to undergo serological screening in accordance with protocols established by the Brazilian Ministry of Health^17^. None of the individuals tested positive by serology or presented signs or symptoms compatible with human visceral leishmaniasis.

## RESULTS

Among the blood samples examined, 70 presented thrombocytopenia, seven eosinophilia, and 55 anemia. In addition, three cases of infection by *Piroplasma* spp., one case by *Anaplasma* spp., 26 cases by *Ehrlichia canis*, and five cases by *Hepatozoon* spp. were detected (Table 1).

**Table 1:** Hematological alterations in dogs from Monte Negro municipality, Rondonia State, Brazil, 2024-2025.

Parameter	Number of cases	Percentage (%)
Total dogs evaluated	450	100%
Thrombocytopenia	70	15.6%
Eosinophilia	7	1.6%
Anemia	55	12.2%
*Piroplasma* spp.	3	0.7%
*Anaplasma* spp.	1	0.2%
*Ehrlichia canis*	26	5.8%
*Hepatozoon* spp.	5	1.1%
Total of hematological findings	167	37.2%

A total of 450 dogs were evaluated in urban areas of Monte Negro municipality, Rondonia State. Seroreactive dogs were identified in neighborhoods locally designated as Sectors 2, 3, and 4, where stratified analysis of serological results was performed.

Among the examined dogs, 45 (10.0%) tested reactive in the TR-DPP^®^ immunochromatographic test. These samples were subsequently analyzed by ELISA, of which 34 (75.6%) were positive for anti-*Leishmania* spp. antibodies. Sector 2 presented the highest number of TR-DPP^®^ reactive animals (n = 25), of which 22 were confirmed by ELISA. Sector 3 presented 11 TR-DPP^®^ reactive dogs, all confirmed by ELISA, whereas Sector 4 presented 10 TR-DPP^®^ reactive dogs, with one confirmed by ELISA (Table 2). 

**Table 2:** Distribution of seroreactive dogs according to urban sectors in Monte Negro municipality, Rondonia State, Brazil.

Sector designation	TR-DPP^®^-reactive dogs (n)	ELISA-confirmed reactive dogs (n)
Sector 2	25	22
Sector 3	10	11
Sector 4	10	1
Total	45	34

Stratified serological analysis was performed only in sectors where seroreactive dogs were identified.

No amplification of the expected *Hsp70* gene fragment was observed in any of the analyzed samples.

The phlebotomine fauna recorded in Monte Negro municipality was composed of six species: *Psychodopygus davisi*, *Nyssomyia antunesi*, *Migonemyia migonei*, *Evandromyia piperiformis*, *Brumptomyia pintoi*, and *Evandromyia saulensis*, comprising five males and 11 females (Table 3). *Nyssomyia antunesi* was the most frequent species, accounting for 50.0% of the captured phlebotomines.

**Table 3:** Phlebotomine species captured in Monte Negro municipality, Rondonia State, Brazil, from February 2024 to April 2025.

Species	Environment	Males	Females	Total	%
*Psychodopygus davisi*	Peridomestic	0	1	1	6.3%
	Forest	1	0	1	6.3%
*Nyssomyia antunesi*	Peridomestic	2	4	6	37.5%
	Forest	0	2	2	12.5%
*Migonemyia migonei*	Peridomestic	0	1	1	6.3%
*Evandromyia piperiformis*	Peridomestic	1	1	2	12.5%
*Brumptomyia pintoi*	Peridomestic	1	1	2	12.5%
*Evandromyia saulensis*	Peridomestic	0	1	1	6.3%
Total	-	5	11	16	100%

Across the sampled environments, most phlebotomines were collected in peridomestic areas, corresponding to 81.3% (13/16) of the total, while forest areas accounted for 18.7% (3/16). *Nyssomyia antunesi* was predominantly collected in peridomestic environments (75.0%; 6), with the remaining specimens identified in forest areas (25.0%; 2/8).

## DISCUSSION

The results of this study should be interpreted in the epidemiological context of VL in Brazil, characterized by marked spatial heterogeneity and complex interactions among reservoirs, vectors, and environmental factors. Since the 1970s, the urbanization of VL has been widely documented and associated with unplanned urban expansion, intensification of migratory flows, environmental changes, and the reduction of natural ecological barriers, favoring the emergence of transmission foci in areas previously considered disease-free^20^.

The identification of 45 dogs reactive in the TR-DPP^®^ test, corresponding to 10% of the evaluated population, suggests previous exposure to *Leishmania* spp. antigens. However, serological cross-reactions with other pathogens commonly found in dogs in Brazil, especially hemoparasites such as *Babesia* spp. and *Ehrlichia canis*, should also be considered, since these agents may induce nonspecific immune responses^21^ ^,^ ^22^. In this study, hematological alterations such as anemia and thrombocytopenia, together with the identification of *Babesia* spp. in blood smears, reinforce this possibility^22^.

Canine infection has been recognized as an important sentinel marker of parasite circulation, frequently preceding the occurrence of human cases, especially in areas of emerging or incipient transmission^3^ ^,^ ^4^. Studies conducted in different regions of Brazil have reported canine seroprevalence ranging from 5% to 15% during the early stages of disease expansion, prior to the consolidation of the domestic transmission cycle and the occurrence of human cases^23^. 

The high concordance observed between the TR-DPP^®^ and ELISA tests, with 34 ELISA-confirmed samples among the reactive dogs, reinforces evidence of previous exposure to *Leishmania* spp. antigens. Previous studies report sensitivity and specificity values above 90% for the combined use of TR-DPP^®^ and ELISA in canine visceral leishmaniasis surveillance^24^. Nevertheless, the absence of molecular detection by PCR in all analyzed samples indicates that seropositivity alone should not be interpreted as definitive evidence of active infection^21^.

Similar findings have been reported in studies conducted in areas of emerging transmission, where seropositive dogs frequently present negative PCR results when peripheral blood is used as the biological sample^23^. This finding may be associated with low parasitemia in subclinical or chronic infections, as well as with the heterogeneous distribution of the parasite in tissues. In canine visceral leishmaniasis, tissues such as spleen, lymph nodes, and bone marrow generally present greater sensitivity for molecular detection^18^.

In addition, the methodological strategy adopted in this study, involving pooled samples containing up to five individuals, may have reduced the analytical sensitivity of PCR, particularly in samples with low parasite burden. Previous studies have demonstrated that pooling samples may reduce the analytical sensitivity of PCR assays due to dilution of target DNA, particularly when pathogen load is low, increasing the likelihood of false-negative results in subclinical infections^25^.

The *Hsp70* gene was selected as the molecular target due to its applicability in the detection and characterization of *Leishmania* species. However, this marker presents lower analytical sensitivity when compared with high-copy targets such as kDNA minicircles, particularly when peripheral blood samples are analyzed. Therefore, the absence of amplification does not completely exclude parasite circulation in the municipality but rather suggests the absence of detectable infection under the analytical conditions employed^18^ ^,^ ^25^.

In the regional context, although Rondonia State has historically been considered an area without autochthonous transmission of canine visceral leishmaniasis, recent evidence indicates geographic expansion of the disease within the state. The reports of autochthonous canine cases in municipalities such as Porto Velho, Ji-Parana, and Cacoal, combined with the identification of competent vectors in urban and peri-urban areas, point to an ongoing process of epidemiological vulnerability^1^ ^,^ ^13^ ^,^ ^14^.

The composition of the phlebotomine fauna identified in this study reinforces this scenario. Studies conducted in different regions of Brazil shows that, even in the absence of high densities of *Lutzomyia longipalpis*, species such as *Migonemyia migonei* and *Nyssomyia antunesi* may act as potential or secondary vectors in anthropized environments^26^ ^,^ ^27^. Recent studies conducted in Porto Velho detected DNA of *L. infantum* in female *Ny. antunesi*, suggesting its possible involvement in the maintenance of the local transmission cycle^17^.

Although the number of phlebotomine specimens captured in this study was limited, possibly due to environmental and operational conditions, the presence of these species indicates an entomological context compatible with potential transmission risk^10^ ^,^ ^14^ ^,^ ^26^.

From a One Health perspective, the combination of canine seroreactivity, presence of phlebotomines with epidemiological importance, and environmental modifications may contribute to conditions favorable for the emergence of visceral leishmaniasis. Integrative studies show that the establishment of the domestic transmission cycle may occur gradually and can be preceded by periods of silent or subclinical circulation among canine populations^11^. Therefore, the identification of seroreactive dogs associated with the presence of potential vectors, even in the absence of molecular confirmation, may constitute an epidemiological warning sign requiring continuous surveillance^3^ ^,^ ^13^ ^,^ ^17^ ^,^ ^23^.

Thus, the findings reinforce the importance of integrated surveillance for visceral leishmaniasis in Monte Negro municipality, including serological and molecular monitoring in dogs, continuous entomological surveillance, and health education activities targeting the local population, as recommended by the Brazilian Ministry of Health^17^. Future studies using larger entomological sampling efforts and tissue samples with greater sensitivity for molecular detection are recommended to better characterize the potential establishment of the local transmission cycle.

## CONCLUSION

The results of this study indicate the absence of evidence of active transmission of *Leishmania infantum* in Monte Negro municipality, Rondonia State, during the evaluated period. However, the detection of seropositive dogs and the presence of phlebotomines as potential vectors in peridomestic environments and forest fragments, despite their low abundance, indicate a potential epidemiological risk, suggesting the potential emergence of an autochthonous focus of visceral leishmaniasis in the Western Amazon.

In this context, strengthening continuous surveillance strategies is recommended, including serological and molecular monitoring of dogs, systematic entomological surveillance, and health education activities directed at the local population. In addition, coordination between local health authorities and research institutions is essential to prevent the establishment of disease transmission and protect vulnerable Amazonian populations.

## Data Availability

The complete anonymized dataset supporting the findings of this study is included within the article itself.
